# Progress towards the achievement of MDG4 in the Commonwealth of Independent States: uncertain data, clear priorities

**DOI:** 10.1186/1478-4505-8-5

**Published:** 2010-02-12

**Authors:** Adriano Cattaneo, Ilkhom Gafurov, Tamara Bomestar, Marianna Bacci, Sanjiv Kumar, Dragoslav Popovic, Giorgio Tamburlini

**Affiliations:** 1 Health Services Research, Epidemiology and International Health, Institute for Maternal and Child Health IRCCS Burlo Garofolo, Via dell'Istria 65/1, Trieste, Italy; 2 UNICEF Regional Office for Central and Eastern Europe and Commonwealth of Independent States, Geneva, Switzerland

## Abstract

Data on under five mortality in the twelve countries of the Commonwealth of Independent States show important fluctuations over time due to variations in quality of data, definitions of neonatal deaths and methods of mortality estimation. Despite the uncertainties regarding mortality trends, the analysis of health and social information from different sources offers clues to identify priority areas and key strategic directions for accelerating the achievement of the 4^th ^Millennium Development Goal. Neonatal deaths represent from 40% to over 50% of under five deaths in all these countries. Maternal mortality was above 50 per 100,000 in 2005, despite the good coverage with antenatal care and births assisted by skilled birth attendants. The scanty information on quality of perinatal care indicates widespread substandard care at all levels. Stunting in children under five is above 10% in ten out of twelve countries and coexists with emerging overweight. Exclusivity and duration of breastfeeding fall short of what is recommended. There are important inequalities in child and maternal mortality, malnutrition and access and use of health services within countries. Taken as a whole, the available information clearly indicates that priority should be given to improvement of the health of women in reproductive age and of the quality of perinatal care, including the establishment of reliable data collection systems. To achieve this, action will need to focus on strengthening the capacity of the health system to improve the technical content of service provision, and on improving access and appropriate use of services by the most disadvantaged groups. The involvement of other sectors will be necessary to improve reproductive health and nutrition at community level and to tackle inequity. Comparisons between countries with similar socioeconomic background but different health policies seem to indicate that gradual progression towards universal coverage with essential health care through a national health insurance system is associated with larger reduction of child mortality than troubled transition towards a privatized and unregulated health system.

## Introduction

The Commonwealth of Independent States (CIS) countries shared the same socio-political system for most of the 20^th ^century. They inherited from USSR a health system characterized by universal access, an often plethoric network of health facilities and abundant staff. Following the collapse of USSR, the already poorly maintained health infrastructure got further worse, supplies became increasingly problematic, and the salaries of health professionals lost most of their purchasing power, while the benefits ensured by the previous generous welfare system were progressively reduced. From 1991, CIS countries followed distinct transition paths and now differ substantially in terms of economy, political system, social policy and health system. We analyzed the available data on child mortality as well as health and social information from different sources in an attempt to identify priority areas and key strategic directions for accelerating the achievement of 4^th ^Millennium Development Goal (MDG4). For mortality data, we chose to use the data reported in UNICEF State of the World Children (SOWC) from 2005 to 2009 [1-5]. The analysis of other data on mortality, morbidity, nutrition, health care (structure, access, use, coverage, quality), and on environmental and social determinants of child health, was based on other international sources such as the TransMONEE database of UNICEF http://www.unicef-irc.org/databases/transmonee/ and related publications [6-9], the WHO World Health Reports and World Health Statistics [10-15], the UNDP World Development Report [16], the WHO/EURO/Health-for-all (HFA) database http://www.euro.who.int/hfadb, and the reports of Demographic and Health Surveys (DHS, http://www.measuredhs.com/) and Multiple Indicators Cluster Surveys (MICS, http://www.unicef.org/statistics/index_24302.html) carried out periodically in CIS countries.

## Discussion

Table 1 illustrates, based on UNICEF SOWC from 2005 to 2009, the pattern of child mortality (U5 MR) over the period 2004-2008 in the twelve CIS countries and the rate of progress since 1990. Based on the latter, five countries (Georgia, Tajikistan, Uzbekistan, Belarus and Ukraine; the first three with an U5 MR well over 30 per thousand) may not be able to achieve the 2/3 reduction by 2015 envisaged by MDG4, while Armenia and Azerbaijan have almost achieved the target and the remaining countries show promising improvement. The patterns of U5 MR show dramatic changes in some countries over the last 5 years: Azerbaijan, for example, had a sudden drop in 2007; the same occurred to Kyrgyzstan, Kazakhstan and Turkmenistan in 2006. Among countries in Central Asia, only Tajikistan showed a smooth pattern of reduction over the last five years, while all showed stagnation or even reversal from 1990 to 2004. Among countries with low U5 MR, there is a common pattern of rather slow improvement, but with important fluctuations in Ukraine and Belarus. Georgia, Armenia and Moldova show consistent improvement, but progress was much greater in the last two countries.

**Table 1:** Under five mortality rates in CIS countries in 1990 and between 2004 and 2008, as reported by UNICEF SOWC.

Country	1990*	2004	2005	2006	2007	2008	Progress (%) 1990-2008*
Armenia	56	32	29	24	24	23	59

Azerbaijan	98	90	89	88	39	36	63

Belarus	24	11	12	13	13	13	46

Georgia	47	45	45	32	30	30	36

Kazakhstan	60	73	73	29	32	30	50

Kyrgyzstan	74	68	67	41	38	38	49

Moldova	37	28	16	19	18	17	54

Russian F.	27	21	18	16	15	13	52

Tajikistan	117	118	71	68	67	64	45

Turkmenistan	99	103	104	51	50	48	52

Ukraine	25	18	17	24	24	16	24

Uzbekistan	74	69	68	43	41	38	49

* according to SOWC 2009

We made an attempt to identify possible associations of the reported trends in U5 MR with socioeconomic trends. We carried out a multivariate statistical analysis on variations over time of the U5 MR and of the social and economic indicators listed in Table 2[16]: there was apparently no correlation. There is, however, a non surprising inverse correlation between GDP per capita and U5 MR. A comparison between countries, such as Georgia and Moldova, which shared a very similar socio-economic profile at the beginning of the '90s but then followed very different paths in terms of political stability and economic and social policies, provides some hints on factors possibly involved in the differential progress towards MDG4 [17]. While Moldova moved gradually towards almost universal coverage with essential health care through a national health insurance system, Georgia had a troubled transition towards a privatized and unregulated health system. Between 1990 and 2005-06, the U5 MR fell by 53-55% in Moldova and by 32-36% (or by 4% if the 2004-05 data are used) in Georgia, despite a higher GDP and ODA in the latter (Table 2). Other factors that may be associated to child mortality, such as maternal education, are unlikely to play a major role since in all these countries, with the possible partial exception of Tajikistan, women's education is high. Maternal education could possibly play a role in minority groups, but there are no data to provide an evidence-based answer.

**Table 2:** Selected social and economic indicators in CIS countries, ranked by Human Development Index[16].

Country	HDI rank (2005)	Population (2005)	Life expectancy at birth (2000-2005)	Total fertility rate (2000-2005)	GDP/PPP (USD) (2005)	Gini index (2002-2004)	ODA (USD p.c.) (2005)	Health expenditure in USD/PPP p.c. and % of GDP (public/private) (2004)
Belarus	64	9.8	68.4	1,2	7918	29.7	5.5	583 (3.7/2.3)

Russian F.	67	144.0	64.8	1,3	10845	39.9	-	427 (4.6/1.6)

Kazakhstan	73	15.2	64.9	2,0	7857	33.9	15.1	264 (2.3/1.5)

Ukraine	76	46.9	67.6	1,2	6848	28.1	8.7	427 (3.7/2.8)

Armenia	83	3.0	71.4	1,3	4945	33.8	64.1	226 (1.4/4.0)

Georgia	96	4.5	70.5	1,5	3365	40.4	69.2	171 (1.5/3.8)

Azerbaijan	98	8.4	66.8	1,7	5016	36.5	26.6	138 (0.9/2.7)

Turkmenistan	109	4.8	62.4	2,8	3838	40.8	5.8	245 (3.3/1.5)

Moldova	111	3.9	67.9	1,5	2100	33.2	45.6	138 (4.2/3.2)

Uzbekistan	113	26.6	66.5	2,7	2063	36.8	6.5	160 (2.4/2.7)

Kyrgyzstan	116	5.2	65.3	2,5	1927	30.3	52.1	102 (2.5/3.3)

Tajikistan	122	6.6	65.9	3,8	1356	32.6	37.1	54 (1.0/3.4)

HDI: Human Development Index; GDP: Gross Domestic Product; PPP: Purchasing Power Parity; ODA: Official Development Assistance; p.c.: per capita.

A look at the causes of childhood deaths gives further insights. Neonatal causes contribute to 50% or more of U5 MR in six countries and to over 40% in all countries. Prematurity is the first cause of neonatal death in all countries. However, while in some countries congenital diseases come second, in other (Armenia, Azerbaijan, Kazakhstan, Kyrgyzstan, Tajikistan and Turkmenistan) asphyxia and infection come second and third. Neonatal mortality rates (NMR) are high in all countries of Caucasus and even more in Central Asia, in spite of decreasing fertility and high reported coverage with antenatal care and births assisted by skilled personnel [11]. The high NMR is consistent with high maternal mortality ratios (MMR). This was estimated, in all CIS countries, to have decreased only from 58 (2,780 deaths) to 51 per 100,000 (1,810 deaths) between 1990 and 2005 [18], thus jeopardizing also the achievement of MDG5. The high MMR and NMR point to problems in the quality of care for pregnant women and newborns. Unfortunately, there is little published information on quality of care. An evaluation of a 5-year project aimed at improving the quality of maternal and infant care carried out in two districts each in Kazakhstan, Kyrgyzstan, Tajikistan, Turkmenistan and Uzbekistan in 2000, found that the quality of care in these districts was generally improving, though still inadequate, for healthy infants and newborns, but was far from satisfactory for sick mothers and sick and premature neonates [19]. Quality of care was shown to be often suboptimal also for infants and older children. A study carried out in 17 hospitals in Moldova, Kazakhstan and the Russian Federation in 2002 found that unnecessary hospital admissions and lengthy stays were common, while most children received excessive and ineffective treatment [20].

Nutrition is an important contributing factor to child mortality, particularly in its post-neonatal component. In CIS countries, the U5 MR correlates strongly with the rates of moderate plus severe stunting (r = 0.76) and underweight (r = 0.81) [21]. Stunting is above 10% in all countries except Belarus and Ukraine, and reaches 27% in Tajikistan, but in all countries, except Azerbaijan, Tajikistan and Turkmenistan, the rate of overweight is higher than the rate of underweight, and exclusivity and duration of breastfeeding are far from optimal [21]. There is unfortunately very little, if any, information on timely and adequate complementary feeding, while information on micronutrient deficiencies shows that vitamin A deficiency is highly prevalent in Central Asia and Azerbaijan [21]. Only Tajikistan and Uzbekistan have more than 20% of children with less than 50 mcg/l of urinary iodine; Armenia, Georgia, Kazakhstan and Turkmenistan have achieved sustainable elimination of iodine deficiency [21]. Kyrgyzstan and Uzbekistan have a severe iron deficiency anaemia problem; all the other countries fall into the moderate public health problem category.

Finally, data show that there are important inequalities in U5 MR within countries. Where information is available, U5 MR is 50% to over 100% higher in families in the poorest wealth quintile compared to the richest. Similar inequalities occur by gender, urban/rural residence, level of maternal education, and ethnic or language group. They occur at various degrees also for other health indicators, such as maternal mortality and malnutrition, and for indicators of access and use of health services, such as antenatal care and careseeking for pneumonia and diarrhoea.

## Conclusions

A first, clearly emerging issue regards the remarkable fluctuations and sudden drops reported over the years in U5 MR in most CIS countries, and particularly in the Caucasus and Central Asia. These patterns are very unlikely to reflect the real situation and are due to a combination, variable across countries and periods, of factors. First, there are important differences between official statistics produced by national authorities and the estimates based on DHS and MICS, almost invariably yielding higher mortality rates. The main reasons for this difference is incomplete reporting in official statistics; the fact that indirect mortality estimates may have large confidence intervals, particularly in countries with lower child mortality rates, should also be taken into account. To sort out differences between estimates produced from different sources using different methods, UNICEF developed, in coordination with WHO, UNDP and the World Bank, a method that minimizes the errors embodied in each estimate and harmonizes trends along time [22]. Second, mortality data depend on definitions, in particular as far as neonatal mortality is concerned. When countries adopt official WHO definitions, a larger number of deaths are classified as early neonatal rather than stillbirths, with a consequent sudden increase in NMR. This is likely to be, with the consequences of the socioeconomic crisis of the early transition period, among the major factors involved in the apparent stagnation or reversal of U5 MR observed in all CIS countries in the decade following the collapse of USSR. Third, the improvement in perinatal care, resulting in lower neonatal mortality, usually coincides with some improvement in data collection, resulting in higher mortality rates; the combined ultimate result of these improvements may explain the fluctuations observed from year to year.

Is it possible to identify trends and priorities, as well as strategies to accelerate the achievement of MDG4 in CIS countries, even if key data, such as U5 MR, are uncertain? We believe that the analysis of available data does provide some indications on priority areas and strategic issues. First, action is needed to improve neonatal health, starting from policies and interventions to improve the health of women in reproductive age and care around childbirth, particularly for at risk pregnant women and newborns through an efficient referral system. Effective specific interventions to improve the outcome of pregnancy and prevent neonatal mortality can reduce NMR where it is high [23]. Where even post-neonatal mortality is high, as in Central Asia, these interventions should be complemented by other effective interventions [24], in an integrated primary health care system [25]. Second, there is direct and indirect evidence that access to care, generally thought to be adequate in CIS countries, badly needs to be matched with action to improve the quality of care. Tools for the assessments and strategies for the improvement of the quality of paediatric hospital care have already been developed and used in many countries, including some CIS countries [26]. The European Office of WHO has developed and is currently using similar tools to assess and improve the quality of maternal and neonatal care at hospital level. Should they prove effective, they should be used in country-wide quality improvement programmes. Third, cross-sectoral action is needed to address the increasing health inequalities and their social determinants [27]. This will become even more important in the next few years, as the current economic crisis will have an impact on several dimensions of household poverty, including nutrition, and on the capacity of the state to adequately finance social policies, such as health and education [28]. The low rates and duration of exclusive breastfeeding and the double burden of under- and overweight indicate poor family practices that require to be improved also by action at community level [29,30]. In countries where health care has traditionally been left to the responsibility of the state, awareness of rights and responsibilities has to be built in the population through the establishment of civil society groups and non-government organizations that will play a key role in stimulating an informed and active participation of communities [31]. The situation of minority groups and socially disadvantaged population groups need to be carefully monitored and appropriately addresses by focused policies. In CIS countries, health and social protection systems have almost everywhere moved away from the previous model to improve efficiency and reduce cost, but the principle of universal access must be maintained for all basic preventative and curative health services and interventions, starting from maternal newborn and child care.

Finally, there is a clear need to improve the quality of data collection, as well as local monitoring and data analysis. The relative stabilization of U5 MR over the last couple of years may indicate that better and more consistent data collection systems are being developed. Improvements have already occurred, for instance by adopting the international definition of neonatal deaths, but a lot remains to be done. Without adequate information on what is really occurring at all levels of the health system, including the community, it will be difficult for policy- and decision-makers and for health professionals to identify more accurately, at national and local levels, effective combinations of policies, strategies and interventions leading to improved maternal, neonatal and child health. It is encouraging to see that the above strategic directions are consistent with the guiding principles (life cycle approach, cross sectoral action, equity and participation) and the objectives proposed by the WHO/EURO strategy for child and adolescent health and development approved by all member states in 2005 [32], and in particular with the first priority area: maternal and newborn health.

## Competing interests

The authors declare that they have no competing interests.

## Authors' contributions

AC and GT coordinated the collection and analysis of data and had primary responsibility in writing the paper. SK and DP provided the data directly and/or through UNICEF Country Offices, and participated in interpretation of results. IG, TB and MB searched and compiled all the data and participated in their analysis and interpretation. All authors worked on subsequent drafts of the paper and approved the final version of the manuscript.
